# Recent Progress of Rice Husk Reinforced Polymer Composites: A Review

**DOI:** 10.3390/polym13152391

**Published:** 2021-07-21

**Authors:** Mohamed Azlan Suhot, Mohamad Zaki Hassan, Sa’ardin Abdul Aziz, Mohd Yusof Md Daud

**Affiliations:** Razak Faculty of Technology and Informatics, Universiti Teknologi Malaysia, Jalan Sultan Yahya Petra, Kuala Lumpur 54100, Malaysia; mzaki.kl@utm.my (M.Z.H.); saa.kl@utm.my (S.A.A.); yusof.kl@utm.my (M.Y.M.D.)

**Keywords:** natural fiber, rice husk, biocomposites, physicomechanical, thermal behavior

## Abstract

Recently, because of the rising population, carbon overloading, and environmental distress, human beings have needed to increase awareness and responsibility for the reduction of agricultural waste. The utilization of agricultural waste as a filler material in reinforced polymers is a fascinating discovery. This review paper attempts to study the physical, mechanical, and thermal behavior of rice husk (RH) as a fiber for reinforcing various synthetic polymers, based on recent studies, conducted between 2017 and 2021. It also highlights that advanced modification techniques could further improve the performance of composites by tailoring the physical and chemical substances of the fiber or matrix. The thermal properties, including flame-retardance and thermal behavior, are also discussed. The characteristics of the fiber–matrix interaction between RH and the polymer matrix provide essential insights into the future-ready applications of this agricultural waste fiber. The way forward in researching RH polymer composites is finally reviewed.

## 1. Introduction

Disposing of waste is problematic, and the most significant challenge today is to find novel ways to utilize these residues. Between 2003 and 2013, Cherubin et al. [1] reported that residues such as rejected crops in the form of leaf litter, straws, sawdust, forest waste, leaves, weeds, and other by-products surged by approximately 33%, as a percentage of the total product. Moreover, agriculture waste reached more than 5 billion Mg in 2013, whereby 47% of the leftover residues were from the Asian continent, followed by America (29%), Europe (16%), Africa (6%), and Oceania (2%) [1]. Landfilling is the primary option to treat this waste; however, issues related to air quality, global availability of land, and greenhouse gas releases, such as methane and leachate, have come to the fore [2,3,4]. Other options are to develop new processing techniques for a higher utilization rate of residues [5].

Malaysia is surrounded by the Straits of Malacca and the South China Sea, and enjoys typical tropical weather, with proximity to water, which gives this country a quite humid, hot [6], and rainy climate throughout the year, with a temperature range from a mild 20 to 30 °C [7]. It is located near the equator and blessed with natural resources, such as crop biomass [8,9,10], hydro, and solar power. In the 1990s, roughly 4.2 million tonnes of vegetable residue and 2.3 million tonnes of livestock waste were produced in Peninsular Malaysia [11]. Table 1 shows the production of agricultural waste in Malaysia in 2007, which was generated from the production of palm oil, rice, rubber, coconut, sugar cane waste, forest products, and municipal waste [12]. Malaysia is an ethnically heterogeneous country and has experienced a radical growth in population. Currently, it is focused on a self-sufficiency policy in rice and paddy production, and this is the country’s primary staple food and food crop. Increasing demand for rice production has significantly increased the waste from rice husk and straw.

Rice (*Oryza sativa* L. genus) is the primary source of daily food intake and has become the world’s second most important cereal crop sector due to the demand of billions of human beings. In 2019, approximately 756 million metric tons of rice were produced globally, and 90% of the total output came from Asia [13]. In Malaysia, about 700,000 hectares of paddy are planted on the extensive agricultural land, yielding more than 800,000 tonnes of rice husk (RH) and stalk waste annually [14]. These wastes should never be burned, due to various reasons, such as the ashes, harmful gases, and fumes that contribute to air pollution [15].

Typically, the RH can be used as biochar, extracted silica, or husk itself. In general, RH is a hull to protect seeds or grains. It is formed from rigid materials, is water-insoluble, and is abrasive, with a high level of cellulose–silica structures. The exterior of the hulls consists of silica covered with a cuticle, with a small amount of silica content at the innermost epidermis.

Recently, several attempts have been made to utilize these waste materials in composite structures. The study of RH as a filler has been of interest to researchers since the 1970s. This paper presents a compressive review of the physical, mechanical, and thermal durability of RH composites between 2017 and 2021, and it details the knowledge gaps that need to be filled in the respective research areas. Furthermore, it discusses the potential of RH composites to be used in photonics, construction materials, and automotive and furniture applications, based on their strength and thermal characteristics.

## 2. Tensile Strength of RH Composites

The exploitation of RH residues in biocomposites offers multiple advantages, for example, reducing the relative amount of constituents derived from synthetic polymers, such as resin polymers and some additives. The tensile strength is mainly used to evaluate the strength behavior of a composite material. The behavior of composites is dependent upon the filler type, matrix material, concentration, size, dispersion, and the adhesion between the filler and the matrix material. Various studies have been conducted on the variation of tensile properties of RH-reinforced composites at different filler loadings using different types of matrix materials as polymer matrices, as tabulated in Table 2. However, they can be classified into four groups: matrix modification, filler treatment/modification, and hybridization.

Using RH as a reinforcement has offered significant enhancements to the tensile properties of composites, as reported by Abdulkareem et al. [16]. They clarified that the Young’s modulus of RH/waste polystyrene (PS) composite increased with increasing the RH content; up to 40 wt % compared to pure PS. A similar improvement in strength was also discovered by Zafar et al. [17] when studying an RH reinforced polypropylene (PP) matrix composite. The maximum tensile strength was achieved at 5 wt % of RH loading, with the size of the RH filler being 355–500 micron. In contrast, this contradicted the findings mentioned by Zhang et al. [18], where increasing the RH filler to 70 wt % decreased the tensile properties due to fiber agglomeration in the matrix. Zhang et al. [19] also studied the tensile strength of RH in a high-density polyethene (HDPE) matrix for different RH loadings, and the best level of tensile strength was attained at 40 wt % loadings. This was the result of the uniform distribution of RH in the matrix, making the matrix tightly wrap the RH, and thus improving the interface bonding. In another study, a unique type of RH, called hydrochar, was reinforced with polylactic acid (PLA) as the matrix, and it was observed that the tensile modulus improved from 2.63 GPa in virgin PLA to 4.24 GPa after blending with hydrochar. Xue et al. [20] used the ball milling technique to enhance the filler–matrix interaction by refining the particle size. They found that the tensile strength increased 44% compared to unmilled RH.

While studying the performance between unfilled and filled epoxidized natural rubber (ENR) with RH ash, it was shown that the filled ENR provided a higher tensile strength than the unfilled ENR. A study of the differences in mechanical properties between RH ash filler and high-purity silica in an epoxy matrix composite by Fernandes et al. [21] found that similar characteristics were observed. They claimed that RH ash could replace silica with little loss of desirable properties. Pongdong et al. [22] indicated a similar conclusion, whereby they found that RH ash filler exhibited a similar reinforcement compared to conventional siliceous earth for epoxidized natural rubber matrix composites.

In order to reduce the level of alkalinity of the pore water in a synthetic polymer, matrix modification has been promoted. This method can typically enhance the durability of the fiber–matrix interaction by using cementitious materials. The tensile strength of the RH reinforced hybrid recycled HDPE/polyethylene terephthalate (PET) composites was optimum at 70 wt % of filler loading, as reported by Chen et al. [23]. In a similar study, Raghu et al. [24] used maleic anhydride grafted polypropylene (MAPP) and m-isopropenyl α–α-dimethylbenzyl-isocyanate grafted polypropylene (m-TMI-g-PP) as coupling agents. They found that the tensile properties of the RH/PP composites were better than the control samples. They observed that at 50 wt % RH loading, the tensile strength increased by 52% as compared with another type of filler, which were encouraging results.

Several researchers evaluated the improved tensile strength properties between RH and the matrix resin using surface modification techniques such as esterification, silane treatment, fiber mercerization, or fiber surface modification. For example, Rajendran et al. [25] treated RH with ultraviolet-ozonolysis and found that the treated RH composites improved the tensile strength by 5% compared to the composite with untreated RH. Bisht et al. [26] used a mercerization treatment on RH flour and studied the effect on the tensile strength of RH/epoxy composites. The tensile strength of the composite improved by 36% with treated sodium hydroxide (NaOH) solution, by up to 8%. Santiago et al. [27] compared the tensile strength of RH powder in a recycled acrylonitrile butadiene rubber/PP hybrid matrix between a silane treatment and anhydride (AC) treatment of the fillers. Again, the AC treatment exhibited better tensile strength compared to the silane treatment.

Zhang et al. [28] analyzed an extracted RH biochar reinforced HDPE composite at different pyrolysis temperatures using injection moulding. The best tensile properties of the composites were obtained in the temperature range of 500–600 °C, due to their outstanding physical interlocking structures. A similar pyrolysis of RH work was conducted by Moreno et al. [29]. It was shown that the increased RH content in the PP matrix led to a proportional decrease in the tensile strength. However, the decrease in tensile strength was less significant for the pyrolysis composites, as verified by the fracture surface.

In addition, Boonsuk et al. [30] mentioned that an alkaline treatment with 11% *w/v* of NaOH removed the hemicellulose layer of RH and offered an outstanding tensile strength improvement, by a factor of 220%, compared to the neat thermoplastic starch. It improved the matrix-filler load transfer capabilities due to the loss of hemicellulose and the rougher outer surfaces after alkaline treatment. By contrast, the combination between untreated and 5% RH loading in a flexible polyurethane (PU) was found to have the best tensile performance of the composites [31]. The treated RH with 10% *w/v* NaOH adversely affected the surface of the filler and decreased the tensile behavior.

Some researchers fabricated hybrid RH composites by combining two or more different types of fillers within a common matrix. For example, Shubbar [32] evaluated the tensile properties of RH combined with fumed silica nanopowder in an epoxy matrix. The tensile properties increased by 50%, just by adding 5 wt % RH, compared to the sample with pure resin. Furthermore, Awang et al. [33] evaluated RH combined with titanium oxide (TiO_2_) and zirconium oxide (ZnO) in the PP matrix and proposed that the addition of TiO_2_ gave a higher tensile strength and Young’s modulus compared to the addition of ZnO. From the scanning electron microscope (SEM) images, they concluded that this higher tensile strength was due to a better interaction between the matrix and the RH particles. Additionally, Kumar et al. [34] assessed a combination of RH/bauhinia-vahilii-weight/sisal filler with epoxy as the matrix and concluded that the addition of RH improved the tensile strength by 34.42% compared to not using the RH filler loading.

The application of RH as a filler in polymer matrix composites increased the tensile strength in all the research that was reviewed in this paper. RH could replace silica and other fillers; however, some of the research showed that the tensile strength increment had a maximum point after a certain amount of RH loading. Some researchers conducted additional studies on the improvement of the interface properties of the filler–matrix, either by surface modification of RH or to the matrix formulation. It is believed that this is the way forward for increasing the usage of RH in polymer matrix composites.

## 3. Flexural Strength of RH Composites

In order to characterise the bending properties of the composite material, the most classical test used to characterize this behaviour is the flexural test (three or four points). A study by Zhang et al. [18] reported that the bending strength of a RH biochar/HDPE composite reached 53.7 MPa, which was far beyond wood–plastic composites. It was indicated that the biochar behaved as a rigid grain and locked the movement of a particle in the polymer chains. Hidalgo-Salazar et al. [35] analyzed a RH-reinforced PP composite and recorded an increase of 75% in flexural strength for the RH/PP composite compared with neat PP. They attributed the increase in bending properties to the stiffening effect of RH in the PP matrix. Singh et al. [36] also measured the flexural strength of a fully recycled RH-reinforced corn starch matrix composite and mentioned that the maximum flexural strength was 19.60 MPa for a RH/corn starch composite with 15 wt % RH content.

Flexural modulus is a material characteristic that is significantly influenced by the morphology and crystallinity of polymers. In particular, the heterogeneous structure of the surface layers is important for high values of flexural modulus. Using a compatibilizer, Chen et al. [23] used an ethylene-glycidyl methacrylate (E-GMA) copolymer as a compatibilizer between recycled HDPE and recycled PET, and maleic anhydride polyethene (MAPE) as a coupling agent between the filler and matrix. They reported an increase in flexural strength of 62% with the increase of RH concentration in the polymer blends of recycled HDPE and recycled PET. It was discovered that the use of a compatibilizer increased the strength of the RH composite with the matrix blend. The coupling agent also improved the flexural strength of the RH/PP composites, and an increase of 46% was reported by Raghu et al. [24]. Moreover, when comparing the effect of silane coupling and compatibilizer MAPE on interfacial adhesion properties in RH/HDPE composites, Sun et al. [37] found that the bending strength and flexural strength were improved by 11.5% and 40.7%, respectively. It was observed that the flexural modulus increased with the increase in RH and the technical cellulose fiber amount. It was obvious that the flexural modulus reached higher values at higher quantities of cellulose fibers (20–30 mass%). Furthermore, there was no positive effect on the flexural modulus with a variety of plasma surface treatments of technical cellulose fibers or grafted maleic anhydride (PLA-g-MAH/PLA/30CeF). The smallest effect on the flexural modulus was noted for ozone-treated fillers [38].

Kumar et al. [34] reported an increase of 33% in the flexural strength for RH/bauhinia-vahilii-weight/sisal epoxy composites compared to unfilled composites at all filler loadings. The effects of hybridized RH with groundnut shell (GNS) reinforced with PP were obtained by Guna et al. [39]. The maximum flexural strength of the hybrid composites was obtained with a 20/60/20 GNS/RH/PP ratio, which was 40% higher than the non-hybrid composites. This could suggest that a higher loading of small fillers was inclined to extensive delamination, and the misalignment of the filler in the matrix thus decreased the strength properties.

## 4. Impact Strength of RH Composites

Singh [36] reported that the impact energy of RH/corn starch composites increased with the increase of the amount of RH content. The impact strength reached 0.362 J for composites with 15 wt % RH content.

The mercerization of fibers improved the impact strength, and Bisht et al. [26] reported that the impact strength of RH flour–epoxy composites were highest at 8% NaOH concentration. The reason for the increase of the impact strength was due to the mercerisation treatment, which improved the adhesion between the matrix and fiber by way of removing the voids on the surface of the untreated RHs. Surface modification by silane treatment of a PVC matrix in RH–PVC composites also increased the impact strength to 44%, as reported by Petchwattan et al. [40].

The use of coupling agents, as studied by Raghu [24], showed that the impact strength of RH–PP composites decreased with increasing filler loadings. Jiang et al. [41] explored the possibility of reinforcing RH–PVC composites with basalt fibers (BF) and found a noticeably increase in impact strength, whereby the BF acted as a reinforcing agent and strengthened the mobility of the matrix chains. Additionally, the aspect ratio of BF was higher than RH, thus the shift of the stress from the matrix to the fiber was more effective.

## 5. Water Diffusion Behavior of RH Composites

The water diffusion behavior of fiber-reinforced composites is dependent on the relative mobility of penetrants between the water molecules and polymer parts. In general, this obeys Fick’s diffusion theory, and three classes of diffusion can be determined [45,46]. The measurement of the kinetic diffusion mechanism was evaluated based on Fick’s theory and the fitting of experimental values, as follows:

(1) $$log\;log\;\left(\frac{M_{t}}{M_{\infty }}\right)=log\;log\;k+n\;log\;log\;t$$

where *M_t_* and *M*_∞_ are the water absorption at time *t* and the saturation point, respectively. *k* and *n* are constants.

The diffusion mechanism is reflected in the value of *n*. When the rate of diffusion of the infiltrate is less than the polymer part, Case I of the Fickian diffusion mechanism is obtained. For this case, the value of *n* = 0.5, where the saturated condition corresponding to a time is rapidly gained and conserved inside the composite [47]. However, when *n* = 1.0, this indicates that the diffusion activity is faster than the relaxation process [48]. The mechanism is distinguished by the progressive barrier between the bulging outer part and the inner glassy part of the synthetic polymer. In Case II, an equilibrium penetration diffusion is reached at a constant velocity. The non-Fickian is justified at a 0.5 < *n* < 1.0 diffusion mechanism and does not obey the Fickian laws. At this condition, Melo et al. [49] used a Langmuir-type model to closely interpret the physical phenomenon of water absorption relaxation of natural fibre composites. In some cases, when n is larger than 1, it is known as Super Case II kinetics [50]; however, when *n* < 0.5, this can be classified as ‘Less Fickian’ behaviour.

Table 3 summarises the water absorption kinetics of an RH-reinforced synthetic polymer. Chen and Ahmad [51] reported that the water absorption and swelling showed a linear increase with the increase of RH content. The higher water absorption and swelling with higher RH fiber content were due to the hydrophilicity of RH. This finding agreed with the finding of Abdulkareem et al. [16], where it was observed that the percentage of water absorbed increased with the addition of RH. Abdulkareem et al. [16] attributed the increase in water absorption to the pores and gaps in the RH structure. A different mechanism was observed in the epoxy matrix by Shubbar [32], whereby it was reported that due to the swelling of the composite as a result of water absorption, the epoxy matrix cracked, which in turn generated a capillary effect and caused further water absorption.

The RH was found to be better in terms of its water absorption properties when it was compared with other types of fillers. Muthuraj et al. [64] found that composites containing RH showed lower water absorption compared to other types of fillers, such as wheat husk, wood fibers, and textile waste. This observation was explained by the higher hydrophobicity of RH compared to other fillers. Yusuf et al. [65] compared composites containing RH with composites containing bamboo stem fiber. They found that composites with RH were better in terms of their lower water absorption and swelling thickness due to the lower affinity of RH to water. Sheykh et al. [52] compared RH and bagasse ash in an HDPE composite. The RH–HDPE composite was found to have lower water absorption and thickness swelling properties. This was due to the lower accessible -OH group on the surface of RH compared to bagasse fibers. Mohamed et al. [55] compared the water absorption properties of different contents of hybrid kenaf–RH in a polypropylene composite. Similarly, other researchers found that a higher RH content exhibited lower water absorption properties. This is because kenaf has larger voids and has more hydroxyl groups that can interact with water.

Antunes et al. [66] studied the ability of RH panels (to be used as wall panels) to absorb and desorb moisture using the moisture buffer value test. They found that the higher RH content panels had a better ability for absorbing and desorbing moisture compared to panels with a lower content of RH, which makes them excellent for high humidity applications.

**Figure 1:**
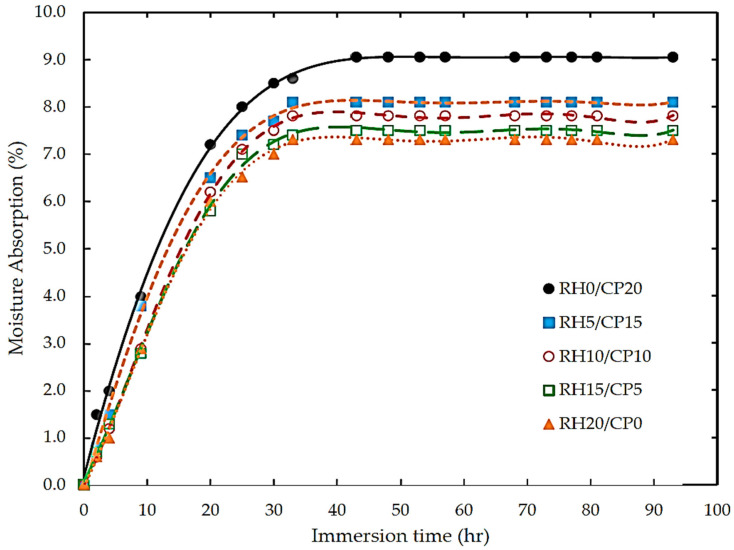
Typical of water absorption plots of RH and cocopeat ABS polymer blend composites.

Akindoyo et al. [58] presented that all composite structures massively absorbed more water than neat PLA due to a natural fiber composite, which contained a higher abundance hydroxyl groups and easily interacted with water molecules. The increase in water absorption was higher in the reinforced blends, which could be credited to the water uptake properties of natural fibers. In general, all the composites conformed with Fickian’s law, where there was an initial rapid water uptake before reaching a saturation plateau region, with further increases in the soaking period. The effect of nano-silica particles extracted from RH on the water absorption characteristics was evaluated by Daramola [59]. An enhancement of the moisture absorption resistance of a nano silica-reinforced HDPE composite was observed at a lower particle weight fraction. However, increasing the filler loading resulted in an increase in the void content, interfacial bonding, and exposure surface between the filler and blend. Similar work was also reported by Hamid et al. [60]. In contrast, they reported that the silica concentration had no significant effect on the water moisture kinetic. Additionally, a nano-silica crystalline composite offered a higher water resistance than a nano-silica amorphous coupon. Both composites had a more hydrophobic resistance compared to epoxy resin. Furthermore, Norhasnan et al. [62] evaluated a hybridized RH/coco peat reinforced ABS, which showed reduced water-resistance biocomposite structures. Figure 1 shows the moisture absorption behaviour of the RH/CP reinforced ABS, and a maximum water kinetic behaviour for 20 wt % of coco peat composite composition was found, due to the higher hydrophilicity of the coco peat particle.

Fiber surface treatment and matrix modification improves the water absorption properties of RH composites. This was confirmed by several kinds of research that used NaOH and silane treatments on RH and coupling agents on the matrix. Huner [53] used 10% NaOH, while Nabinejad et al. [67] used 5% NaOH. Both found that NaOH decreased the water absorption of the RH–PP composite. NaOH treatment caused the surface of the RH to be polar. The same result was also produced by silane treatment of RH. Water absorption decreased by up to 38%, as reported by Petchwattana et al. [40], due to the silane reacting with free OH groups and due to the elimination of voids. Huner [53] reported that the tendency for reaction was lower than NaOH, causing the water absorption rate for silane treated composite to be lower. The use of MAPP decreased the water absorption rate due to the decrease of micro gaps in the interface, as a result of enhanced bonding between the filler and matrix [53]. The comparison between NaOH treatment and UV/O_2_ treatment by Rajendran Royan et al. [25] showed that the NaOH-treated RH exhibited higher water absorption properties. The reason for this was due to the dry treatment with UV/O_2_, where the RH was not soaked in any liquid and as a result, there was no fiber swelling that could give access to water in the reactive region. Saidi et al. [54] used a titanate coupling agent for a RH–PVC composite. Titanate coupling improved the interfacial adhesion between the RH and PVC matrix, preventing the diffusion of water molecules. As a result, the water absorption was reduced by 26%.

Researchers have also used hydrophobic materials to increase the water resistance of the composite structure. As an example, Chalapud et al. [61] used a tung oil in RH that was adhesively bonded by a soy protein composite to improve the moisture resistance of particleboard. The impregnation of the composite panel with tung oil decreased its moisture absorption capacity and reduced the volume of voids, as obtained by surface microscopy. Since the oil was hydrophobic, a longer time period was required to reach saturation conditions, and this reduced the water kinetic mechanism, diffusing inside the hydroxyl groups of the RH and matrix to form hydrogen bonding. Moreover, a gamma radiation post-treatment was also employed by Chen et al. [63] and proved that the irradiation process increased the moisture kinetics and swelling effect on the composites. However, increasing the filler content after post-treatment also increased the moisture absorption, due to a huge quantity of carbonyl and hydroxyl groups in the composite, which allowed more molecule water to be diffused via the capillary effect.

## 6. Thermal Properties of RH Composites

The thermal stability studies using differential scanning calorimetry and thermogravimetry analysis conducted by researchers and reported in this review are summarised in Table 4. Many researchers have studied the thermal stability of polymer composites that were reinforced with RH fillers. Some researchers found a positive effect while others reported a negative effect on the thermal stability of RH in polymer composites. For example, the addition of RH ash to LDPE increased the onset degradation temperature and peak melting temperature, as reported by Zulkiple and Romli [68]. This effect also increased with the increasing percentage of filler loading. They explained that the filler acted as an insulator that could delay thermal degradation. A similar effect was also reported by Hidalgo-Salazar and Salinas [35], and Majeed et al. [69] with the addition of RH to PP. Hidalgo-Salazar and Salinas [35] explained that RH contributed to the increase in thermal stability of a PP composite due to the nucleation of spherulites, which increased the crystallinity of the PP matrix. A positive effect on thermal stability can also be seen in the increase of residual weight of a composite with the increase of RH content, attributed to the increase in thermal degradation of the composite due to the lower thermal degradation of RH compared to PP [69]. RH based silica, which is silica obtained from RH, provided a positive effect on the thermal stability of composites to which it was added. This was reported by many researchers, such as Krishnadevi et al. [70] and Tipachan et al. [71]. Functionalized RH ash, for example RH functionalized with 3-aminopropyltrimethoxysilane (3-APTMS) [70], improved the thermal stability of cardanol based benzoxazine composite. This was due to the formation of a complex network structure of silica reinforcement. The addition of silica nanoparticles from RH [71] improved the thermal stability of the PLA composite due to the thermal stability of the silica.

Many other researchers reported the negative effect of the incorporation of RH in polymer composites. In PP matrix composites, the addition of RH decreased the decomposition temperature due to RH being less thermally stable than PP. This was reported by Arjmandi et al. [72]. When studying natural rubber filled with unmodified RH ash, Zeng et al. [73] reported that the thermal stability was decreased due to the presence of pore structures with a high specific surface energy unevenly dispersed, and which when heated caused poor thermal stability. Similarly, the addition of RH to PLA [74] and PBAT–PLA hybrid composite [64] lowered the thermal degradation temperature, due to the lower thermal degradation nature of RH.

Due to the negative effect of RH on the thermal stability of polymer composites, many researchers have investigated various methods to improve the thermal stability, for example by adding flame retardants, by increasing the fiber–matrix interaction, and by hybridization with other fillers. Flame retardants have been successfully used to increase the thermal stability of RH composites. Ammonium polyphosphate (APP) [72] helped to increase the maximum degradation temperature, due to the formation of phosphorus and charred layers that shielded the PP matrix from further degradation. The addition of clay [69,75,76] thermally stabilized the RH composites, because the exfoliation of clay can delay the decomposition process of composites. The silicate layers of clay produced a twisted path for the diffusion of volatiles, which in turn improved the thermal stability of the composite. The use of halogen-free flame retardants [77] increased the thermal stability by generating thermally stable substances such as aluminum oxide, silicon dioxide, and phosphorus-containing compounds. Increasing the fiber–matrix interaction increased the thermal stability of RH composites [78]. For example, the increase of thermal stability of an epoxy–RH composite was attributed to an improvement of interfacial bonding due to the modification of RH using 3-glycidoxypropyltrimethoxy silane (GPS) [79]. The addition of TiO_2_ also increased the thermal stability of PP–RH composites. Awang et al. [33] attributed this to the enhancement of the PP matrix and RH interaction. Another method of increasing the thermal stability of RH composites is hybridization. For example, a combination of RH and walnut shell [36] raised the thermal stability of a corn starch matrix composite due to the thermal stability of cellulose and lignin. The addition of volcanic ash, RH, and treated solid waste [80] was proven to improve the thermal stability of PP.

## 7. Flame Retardance of RH Composites

**Figure 2:**
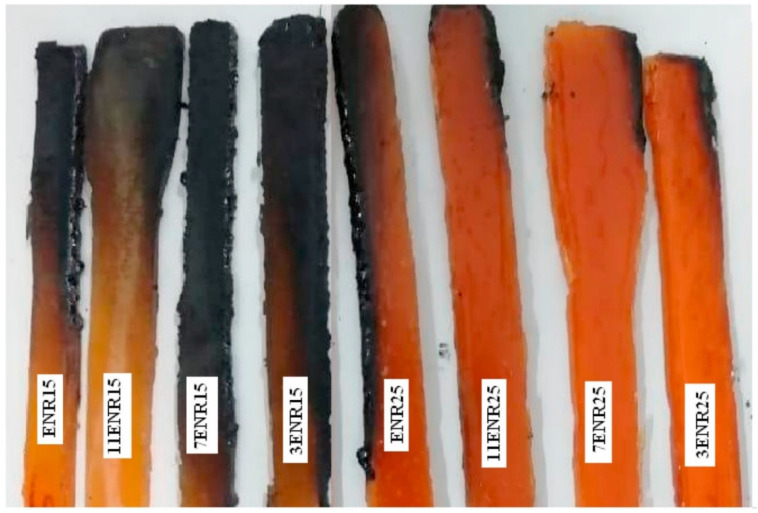
Burn test on RH biocomposites, according to ASTM D 2863 [89].

The addition of RH improved the flame retardant properties of polymer composites. The reason for the improvement of flame retardant properties is mainly the existence of char layers that act as barriers to further degradation. Krishnadevi et al. [70] reported that 20% functionalized RH ash added to cardanol based benzoxazine matrix produced the highest value of limiting oxygen index (LOI) analysis, of 36%. The reason was that the Si-O-Si phase promoted the formation of intumescent char, which could enhance the flame retardance. Other researchers added flame retardants to RH composites to further enhance flame retardancy. Attia and Saleh [83] reported a reduction of peak release heat of up to 33% during a flammability test of styrene-butadiene rubber containing RH silica nanoparticles using a cone calorimeter instrument. This improvement in flammability properties was attributed to the existence of a char layer from the combustion of molokhia extract and that was coated on the RH. The formation of an insulative char layer was also the reason why the addition of clay improved the flame retardant properties of a PVC–RH composite studied by Dutta and Maji [76]. Vu et al. [84] and Vu and Bach [85] studied RH-based silica added to epoxy with the addition of phosphorus-jointed epoxidized oil (DOPO-J-ESO). They found that thick char layers were formed by the combination of a hydroxyl group in the silica and the phosphaphenanthrene group of the DOPO-J-ESO, which improved the flame retardance of the composite. Similarly, the addition of diammonium hydrogen phosphate (DAP) to a polyurethane–RH composite [86] enhanced the fire resistance, due to the existence of residual char when DAP decomposed. This residual char acted as a flame inhibitor and dehydration agent, which further accelerated the formation of char. An increase of char residue was reported by Wu et al. [87] with the incorporation of 30 wt% melamine phosphate modified lignin (MAP-lignin) to 5 wt% RHA in poly(3-hydroxybutyrate-co-4-hydroxybutyrate) (P34HB) matrix composite. The increment of up to 24.3% char residue helped enhance the combustion behavior of the composite. Another type of flame retardant that has been studied is magnesium phytate (Mg-Phyt), which was added to the epoxy–RH composite studied by Xu et al. [88]. The addition of 5% Mg-Phyt to 5% RHA produced a compact silica-rich char layer containing Si-P and P-O-C structures that were thermally stable. Figure 2 represents the flame retardant mechanism evaluation of RH composite samples according to ASTM D 2863 [89].

## 8. RH Composites Applications

RH itself has several benefits, for example, it can be turned into fuel using the pyrolysis process [90]. The highest energy content of RH can be harnessed by this gasification technique, earning substantial combustion products to reduce the use of fossil fuels. RH also contains high levels of lignin and cellulose, which are useful in activated carbon capture [91,92,93]. Wang et al. [91] modified the adsorbent X zeolite from RH for carbon dioxide (CO_2_) capture, prepared using the consolidation of thermal and chemical activation. These modified zeolites improved the mechanism of heat and gas uptake during adsorption and desorption, and they were applicable for long-term capture and separation of CO_2_ from an industrial exhaust gas. Moreover, RH is an advanced material with a high composition of silica that can be used as polishing and cleaning agent [94]. Furthermore, Okoya et al. [95] proposed employing RH biochar as a catalyst in conventional water treatment for removing chlorpyrifos from pesticide polluted water.

Rice husk as a biomass can be converted into various types of carbon products through the process of pyrolysis. One of the applications for this derived carbon is electronic wave absorption, as detailed by Chen et al. [96]. In addition, rice husk has a high silica content and, significantly, offers unique physical and chemical properties, which can improve the thermal and acoustic features of structures. The utilisation of RH in several applications in the construction industry has been discussed in the literature, such as in engineering cementitious composites (ECC) to produce lighter cement [97] or flexible mortar [98]; in resilient mats [99], where it was used to attenuate the impact of sound in buildings; and in rubber-modified asphalt concrete [100,101]. Zhang et al. [97] suggested that replacing fly ash with RH accelerated the hydration process, pozzolanic reaction, tensile strength, and refined pore distribution in lighter ECC. Nano silica fumes extracted from RH were used as an additive mineral to improve the physical and mechanical properties of flexible mortar composites. Significant improvements in resistance to thermal damage, higher strength, and lower permeability compared to the control cenosphere-based lightweight mortar were observed [97]. Furthermore, composite boards made from a combination of RH and recycled rubber were examined by Marques et al. [99]. These panels were tested as the top layer of a floating insulation floor system. They suggested these composite boards mitigated vibrations, improved impact sound insulation, and performed better as floor coatings. In the short-term ageing at high-temperature performance of an asphalt binder modified with a combination of crumb rubber and RH [100,101] indicated that adding crumb rubber and RH enhanced the ageing resistance as compared with the base asphalt binder. In addition, RH can be used as kiln bricks for plastering mortar to control the thermal properties of buildings [102]. De Silva et al. [103] suggested that a combination of cement, sand, and waste RH increased the strength, durability, and thermal performances of the brick. This mortar was used as a refractory material in a furnace application and retained its physicochemical properties at a temperature of more than 1000 °C [103,104].

The application of RH as biodegradable composites that to be used for interior and exterior parts in the automotive industries such as boot/spare tire lining [105] and brake pads [106] was widely examined. Currently, Volkswagen Group motors use a RH reinforced polymer to fabricate the tailgate, double load floor of the trunk, and covering of the roof for the SEAT León car [105]. The panel specimens were successfully tested to support up to 100 kilos of concentrated load and thermally examined in a climatic chamber, to measure the resistance to heat, cold, and humidity. Furthermore, RH was promoted to be used as a friction material, which has been dominated by the asbestos material. This asbestos-based brake pad was banned due to the carcinogenic and hazardous effects on the human body [106]. The husk filler could reduce the rate of wear, improve the friction behavior, and gave a harsh surface roughness, which resembled a commercial brake pad [107].

High-value applications, such as the use of RH in the manufacturing of silica gels, silicon chips, and ingredients for lithium-ion have been discovered. In semiconductor applications, zinc oxide (ZnO) was utilized due to its better ultraviolet absorbing properties compared to other semiconductor materials. However, it tends to agglomerate, which led to poor degradation. A combination of ZnO and RH as a composite material improved photocatalytic activity under ultraviolet (UV) irradiation [108,109]. Furthermore, RH was also successfully synthesized as a microporous activated carbon through carbonizing and activating it with zinc chloride (ZnCl_2_) for a lithium–sulfur battery application. The composite offered a capacity of 426 mAhg^−1^ at a 2C rate, and was suitable for energy storage devices for electric power [110]. Moreover, Suwanprateeb et al. [111] suggested that RH-reinforced epoxy could be used as an embedding material in electrical and electronic applications. Chen et al. [112] successfully produced a low-cost synthesis of SiC whiskers using a combination of RH and graphene. This whisker displayed excellent chemical reaction thermodynamics and outstanding performance for degrading rhodamine B.

**Figure 3:**
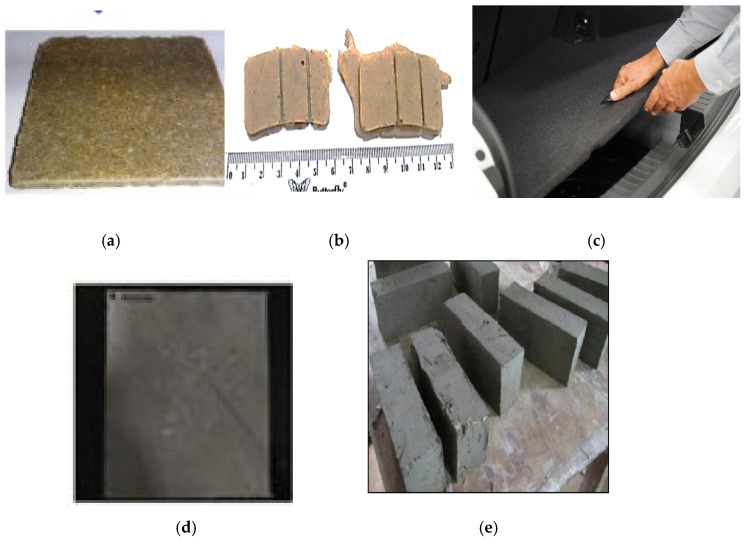
Application of RH biocomposites: (**a**) medium density board [56], (**b**) brake pad [106], (**c**) car trunk [105], (**d**) polymer film [118], and (**e**) refractory mortar [104].

Several studies reported the application of RH fibers in furniture and household appliances, which could support the concept of reducing agriculture waste [113]. Nicolao et al. [13] evaluated hybridized hemp and RH fibers for fabricating medium-density particleboard. They mentioned that this material combination can be considered for applications in building and furniture. Pratheep et al. [114] also verified that high mechanical properties can be obtained with smaller size RH utilization, such as wood powder composite resulting in the reduction of voids and cavities. This wood–plastic composite could be eligible as a decking and flooring fixture. Sadik et al. [115] also proved that the effect of nano-silica extracted from RH in wood plastic composite applications improved the water absorption and thickness swelling of composites. In addition, the thermal stability was slightly enhanced compared with the neat composite. The utilization of RH fibers in composites for food packaging has received a lot of attention from researchers [16,17]. Gupta et al. [116] synthesized a carboxymethyl cellulose (CMC) element from RH fiber to fabricate a biodegradable film. To improve the tensile strength and elongation of the film, the CMC was blended with glycerol and citric acid. Datta et al. [117] also extracted RH fiber to produce crystalline starch phthalate. These thin films offered outstanding mechanical (tensile, tear, stiffness), optical (haze, transmittance), and biodegradation properties, which were suitable for biodegradable food packaging applications. Figure 3 shows the RH fiber that was used in advanced applications.

## 9. A Way Forward: RH Composites

The advantages of RH, including its biodegradability, abundant availability, and low-cost, make this fiber a potential replacement for synthetic polymers. However, the incompatibility between the hydrophilic matrix and hydrophobic fiber reduces the mechanical properties of RH composites. Due to this, chemical and surface treatment techniques to enhance fiber–matrix interfacial adhesion have increased the fabrication cost of these structures. The adoption of optimization techniques such as the Taguchi and response surface method [119,120,121] has great potential and could reduce the cost of experimental setups and the final product itself. In addition, there is also great potential to develop the finite element method software package to compare the mechanical properties of RH composites from experimental works.

The recyclability of waste composite products has been given tremendous attention lately. Many attempts have been made to recycle RH composite waste via manufacturing the product using thermoplastic matrices. The usage of natural resins, including PLA, thermoplastic starch, and corn/soy starch, would help to reduce the environmental impact [122]. These discoveries have great potential to help preserve natural resources in large amounts.

In general, silica is the major constituent of RH ash, which is suitable to be used in thermal insulation, electrical devices, and photonic applications. Inhomogeneous nano-silica, through burning RH in a pure oxygen atmosphere, and costly extraction methods are the major drawbacks of these techniques [123]. New and low cost routes for producing silica, such as by suspending RH in chemical solutions and then precipitating it in an acidic condition for extracting a silica gel, have great potential for further investigation.

Moreover, the degradation rate of RH biocomposite products needs to be examined for the ageing, ultraviolet deterioration, and humidity effects. There is also great potential for evaluating the kinetic degradation reaction rate of RH composite products affected by ubiquitous microorganisms, microbials, and isolated bacteria [124].

## 10. Conclusions

To conclude, recent works on RH-reinforced polymer composites between 2017 and 2021, mainly on physical, mechanical, and thermal behavior have been discussed. In addition, an insightful overview of the future-ready applications has also been given. As highlighted in this review, RH polymer composites have mainly focused on the interface interaction problem between RH and the polymer matrix. Several researchers improved the surface modification of the fiber and matrixes by esterification, silane treatment, and fiber mercerization. Another method is a thorough hybridization with other types of fillers or a mixed blend of the matrices. Both techniques have significantly improved tensile, flexural, and impact strength properties.

Research on moisture kinetics is also related to the fiber surface treatment and matrix modification of composites, due to the high hydrophilicity of RH. Many of the treatments produced improvements of the water absorption properties and swelling effect of RH composites. The thermal stability and flame retardant properties were enhanced by adding flame retardants, increasing the fiber–matrix interaction, and by hybridization. Incorporating flame particles in composites significantly increased the maximum degradation temperature and LOI, however, it reduced the peak release heat.

Potential applications for RH polymer composites have been briefly described. RH in itself has been used as a fuel, for activated carbon capture, polishing particles, cleaning agents, and catalysts for water treatments. As a filler, RH is used in many fields, such as construction, automotive, rechargeable batteries, semiconductors, electricals, electronics, furniture, household appliances, food packaging, and biodegradable films.

The vast potential applications of RH fillers have been described, and we believe that cost reduction should be emphasized in terms of research and product development. We also suggest that the degradation of performance from an environmental and biological organism perspective is an interesting subject to be investigated.

## Figures and Tables

**Table 1:** Crop waste production in Malaysia in 2007 [12].

Types	Quantity (kt)	Source	Source (kt)
**Agricultural waste**			
Oil palm fronds	46,837	Oil palm FFB	81.92
EPFB	18,022		
Oil palm fibers	11,059		
Oil palm shells	4506		
Oil palm trunks	10,827		
Paddy straw	880	Replanting paddy	2375
RH	484		
Banana residues	265	Banana	530
Sugarcane bagasse	234	Sugarcane	730
Coconut husk	171	Coconut	505
Pineapple waste	48	Pineapples for factories	69
**Forest residues**			
Logging residues	2649	Logs	2649
Plywood residues	2492	Plywood	2492
Sawmill residues	1.16	Sawn timber	1418
**Municipal solid waste**			
Organic waste	4653	MSW	6744

**Table 2:** Reported studies of the mechanical properties of RH reinforced polymers.

Matrix	Parametric Study	Tensile Strength (MPa)	Flexural (MPa)	Impact Strength (kJ/m^2^)	References
HDPE	Matrix modification	22.5 ± 0.5	49.6 ± 1.2		Abdulkarem et al. [16]
PP	Filler loading	19.7	39.2		Zafar et al. [17]
HDPE	Filler modification	20	53.7	13	Zhang et al. [18]
HDPE	Hybrid	15.8	25.7	15.2	Zhang et al. [19]
Natural rubber	Filler modification	21.3 ± 0.7			Xue et al. [20]
Epoxy	Filler modification	120			Fernandes et al. [21]
Epoxidized natural rubber	Filler modification	18.5 ± 0.5			Pongdong et al. [22]
HDPE/PET	Matrix modification	22.2 ± 0.1	48 ± 2	3 ± 0.1	Chen et al. [23]
Epoxidized natural rubber	Matrix modification	35	45	22	Raghu et al. [24]
rHDPE	Filler treatment	18.37			Rajendran et al. [25]
Epoxy	Filler treatment	46 ± 1	87 ± 2	2.7 ± 0.1	Bisht et al. [26]
rABS/PP	Matrix modification	21 ± 1			Santiago et al. [27]
HDPE	Filler treatment	26.3 ± 0.50			Zhang et al. [28]
rPP	Filler treatment	28 ± 0.25		3.0 ± 0.5	Moreno et al. [29]
TPS	Filler treatment	2.43 ± 0.25			Boonsuk et al. [30]
PU	Filler treatment	0.25 ± 0.11			Olcay et al. [31]
Epoxy	Hybrid	43			Shubbar [32]
PP	Hybrid	40 ± 2			Awang et al. [33]
Epoxy	Hybrid	30 ± 2	25 ± 2		Kumar et al. [34]
PP	Filler loading	33.2 ± 0.5	39.8 ± 0.3		Hidalgo-Salazar et al. [35]
Corn starch	Hybrid	10.7	19.6		Singh et al. [36]
HDPE	Matrix modification		30 ± 2		Sun et al. [37]
PLA	Matrix modification		5254 ± 25		Běhálek et al. [38]
PP	Hybrid	15.6 ± 0.25	37.6 ± 1.88		Guna et al. [39]
PVC	Matrix modification	51.9 ± 2.54		74.9 ± 5.81	Petchwattana et al. [40]
PVC	Hybrid			5.5 ± 0.80	Jiang et al. [41]
Cassava starch	Filler modification	3.3 ± 0.5			Kargarzadeh et al. [42]
Corn starch	Matrix modification	14.3 ± 1.13			Yap et al. [43]
PLA and PBAT	Matrix modification		10.0 ± 1.0		Spada et al. [44]

**Table 3:** Reported studies of moisture absorption kinetics of RH-reinforced polymers.

Matrix	Parametric Study	M_∞_(%)	Thickness Swelling (%)	Diffusion Coefficients (D × 10^–5^ mm^2^/s)	References
rHDPE	Hybrid	1.8–4.0	4.8–6.8		Chen and Ahmad [51]
HDPE	Filler content	12.0–13.0	8.5–10.0		Sheykh et al. [52]
PE	Filler treatment	3.0–7.0			Nabinejad et al. 2017
Cassava starch	Hybrid	0.5–2.7			Huner [53]
PVC	Coupling agent	0.4–2.4			Saidi et al. [54]
PE	Hybrid	2.5–13.0			Mohamed et al. [55]
Corn starch	Filler content	5.1–11.9			Battegazzore et al. [56]
PVC	Coupling agent	4.2–6.3			Petchwattana et al. [40]
Epoxy	Hybrid	1.2–2.4			Shubbar [32]
rHDPE	Filler treatment	1.7–4.0			Rajendran et al. [25]
rPE	Filler content			2.8–1.6	Abdulkareem et al. [16]
rHDPE/rPET	Hybrid	3.0–9.5	4.0–8.8		Chen et al. [23]
Epoxy	Filler content	0.06–0.17			Fernandes et al. [21]
PLA	Filler treatment	2.5–3.5			Prappuddivongs et al. [57]
PLA/PLB	Blending effect	0.8–5.2			Akindoyo et al. [58]
HDPE	Filler content	0.12–0.28			Daramola [59]
Epoxy	Filler content	0.08–0.13			Hamid et al. [60]
Soy bean	Coating	4.0–11.0		3.2–15.9	Chalapud et al. [61]
ABS	Hybrid	6.9–9.1		1.1–1.4	Norhasnan et al. [62]
rHDPE	Filler treatment	3.7–26.6	0.63		Chen et al. [63]

**Table 4:** Reported studies of the thermal stability of RH-reinforced polymers.

Matrix	Parametric Study	T_g_, °C	5% Weight Loss, °C	10% Weight Loss, °C	T_m_, °C	Char Yield at (600–900 °C), %	References
Cardanol based polybenzoxazine	Filler content	145	319	370	470	47	Krishnadevi et al. [70]
PLA	Matrix modification		335	521		6.1	Tipachan et al. [71]
Organic paraffin	Filler content	51.5			341	7.46	Lai et al. [79]
PLA	Filler treatment				472	3	Chen and Ahmad [51]
PP	Filler content				449.5	3	Arjmandi et al. [72]
Epoxy	Filler treatment	140			156.5		Fernandes et al. [21]
PMMA	Filler content	129			453		Oleiwi et al. [78]
LDPE	Filler comparison				112.9		Zulkipli and Romli [68]
Natural rubber	Coupling agent				447.2		Zeng et al. [73]
PBAT/PLA	Filler content					40	Muthuraj et al. [64]
PP	Filler content				166		Hidalgo and Salinas [35]
rHDPE	Filler content				131		Guo et al. [81]
Corn starch	Filler content		139	166		3.5	Singh et al. [36]
PP	Filler content		356.3	422.4	489.8	9.2	Awang et al. [33]
PP	Filler content				403	22.8	Almiron et al. [80]
PP	Filler content				350	30	Das et al. [82]
PET	Flame retardant effect		234			1.3	Phan et al. [77]
PVC	Clay addition				455	11	Dutta and Maji [75]
PP	Clay addition			311	162.9		Majeed et al. [69]

## Data Availability

The data presented in this study are available on request from the corresponding author.
